# Correlation of Dietary Advanced Glycation End Products with the Hematological and Biochemical Markers of Patients with Chronic Kidney Disease Undergoing Hemodialysis

**DOI:** 10.7759/cureus.6360

**Published:** 2019-12-12

**Authors:** Adamantia Aroni, Sofia Zyga, Maria Tsironi, Demetrios Presvelos, Anastasios Drakopoulos, Maria Ralli, Ioannis Moisoglou, Vasiliki Leventogianni, Georgios Kosmidis, Andrea Paola Rojas Gil

**Affiliations:** 1 Nursing, Faculty of Health Sciences, University of Peloponnese, Tripolis, GRC; 2 Hemodialysis Unit, General Hospital of Molaoi, Molaoi, GRC; 3 Hemodialysis Unit, Nefrologiki Viotias, Livadeia, GRC; 4 Hemodialysis Unit, General Hospital of Kalamata, Kalamata, GRC; 5 Quality & Ongoing Training, General Hospital of Lamia, Lamia, GRC; 6 Hemodialysis Unit, General Hospital of Argos, Argos, GRC; 7 Nursing, Faculty of Health Sciences, University of Peloponnese, Tripoli, GRC

**Keywords:** advanced glycation end products, chronic renal disease, hemodialysis, diabetes mellitus, nutrition

## Abstract

Aim

The advanced glycation end products (AGEs) are among the mechanisms responsible for the pathogenesis and development of chronic kidney disease. The aim of this study was to estimate the dietary AGE intake and to assess its correlation with hematological and biochemical markers of patients with end-stage renal disease (ESRD) undergoing hemodialysis.

Materials and methods

For this study, a structured questionnaire of the exogenous AGEs was developed, whose reliability and validity were evaluated in the pilot phase of the study including 50 participants. The questionnaire was issued to 605 participants (305 ESRD patients undergoing hemodialysis and 300 controls), and a blood sample was obtained through which hematological and biochemical markers were analyzed.

Results

It was noted that patients with ESRD consume large quantities of dietary AGEs not only in absolute values but also in comparison with control subjects (p = 0.001), attributed mainly to the methods of product processing as well as cooking. It was also ascertained that dietary AGEs were correlated (p < 0.005) with fasting glucose and glycated hemoglobin (HbA1c) and with lipidemic profile markers, such as triglyceride, as well as inflammation markers, such as erythrocyte sedimentation rate, ferritin, and C-reactive protein. All the aforementioned markers show abnormally increased levels in patients with ERSD and diabetes compared with healthy subjects.

Conclusion

Patients with ESRD consuming foods favoring AGE formations combined with increased endogenous AGE burden the body with their harmful action. If the specific group of patients adopt dietary habits contributing to the containment or the inhibition of AGE formation, then this would lead to the improvement of their hematological and biochemical markers and in terms of the effects of AGEs on their health is deemed imperative through the creation of consulting and prevention programs.

## Introduction

Advanced glycation end products (AGEs) result from the non-enzymatic reaction of glucose with biomolecules such as proteins, lipids, and nucleic acids (glycation), or are derived from exogenous sources such as nutrition and smoking. They are believed to be responsible for early aging and the pathogenesis of many of diabetes mellitus (DM) microvascular and macrovascular complications, as well as other metabolism-related diseases and other inflammatory or degenerative conditions [1]. AGEs are formed in the presence of hyperglycemia, as well as in diseases that are linked to high levels of oxidative stress, such as chronic kidney disease (CKD). In CKD, the higher levels of AGEs in circulation are due to their increased formation as well as their reduced renal dialysis [2].

Nutrition is a major source of AGEs, with inflammatory and oxidizing properties that are similar to those of endogenous AGEs, and contributes considerably to their overall accumulation in plasma and tissues. The consumption of foods that contain high levels of AGEs plays a key role in the pathogenesis of AGE-related disorders. Factors such as temperature, cooking time, humidity, and pH contribute to promoting and speeding up the formation procedures of AGEs [3]. Thus, various cooking methods can affect the content of AGEs in foods, without necessarily altering the composition of their nutrients. Foods that are rich in fat and proteins, mainly of animal origin, which are cooked at a high temperature for a prolonged time and under dry conditions are the richest dietary sources of AGEs [4].

Even though the contribution of dietary AGEs to the total accumulation of AGEs in the body is fully documented, the role of AGEs in the pathogenesis and progression of chronic diseases such as DM, atherosclerosis, and CKD has not been fully clarified yet. Existing data are mostly derived from intervention studies on experimental animals, primarily on rats, and less on humans [5].

Understanding the mechanisms through which dietary AGEs cause pathological interactions on the kidney is of key importance toward more effective goal setting in CKD treatments, which is associated with lifestyle and improvement in dietary recommendations [6]. The data that have been derived from experimental models, as well as from studies on humans, show that the increased intake of dietary AGEs promotes the progression of kidney failure. Therefore, it would be an officially accepted practice to consider AGEs that are derived from nutrition as a potential treatment goal for kidney diseases and a subject of research studies [7].

This study aimed to determine the dietary intake of AGEs by patients with end-stage renal disease (ESRD) undergoing hemodialysis and to assess its correlation with their hematological and biochemical markers.

## Materials and methods

Survey sample

This is a cross-sectional study including 605 participants. The sample of the survey comprised 305 patients with ESRD who underwent hemodialysis as a method for substitution of their renal function. These patients were recruited from six hemodialysis units in Greece. The control group comprised 300 individuals, of whom 247 were not patients with ESRD but had a metabolic disease, mostly type I or II DM, and 53 were perfectly healthy. The selection criteria for the sample were as follows: (a) age > 18 years, (b) ability to communicate in Greek, (c) satisfactory level of understanding and cooperation, (d) absence of any active infection or neoplasia, and (e) willingness to participate in the survey.

Questionnaire design

To implement the survey's objective, a questionnaire was created to investigate the exogenous sources of AGEs. Drawing on the literature, a questionnaire was constructed, which was divided into three parts. The first part contained questions on sociodemographic data (age, gender, marital status, occupation, family income, place of residence), somatometric characteristics (weight, height, waist circumference, body mass index), and daily habits such as smoking, type and time of physical exercise, type of employment, and sleep. The second part contained questions on family and individual medical history. In the third part of the questionnaire, the respondents were asked to state the frequency and amount of consumption (weekly) of various foods, which had been previously grouped into categories (fruits, vegetables, meat, starchy foods, etc.). They were also asked to state the cooking methods and times.

In the pilot stage of the survey, the reliability and validity of the questionnaire were assessed and documented as it was administered to a group of 50 individuals (13 patients with ESRD and 37 individuals without CKD) from the region of Laconia, Greece. One month after the completion of the first survey, the questionnaire was distributed again to all the respondents since none of them refused to complete the questionnaire once more. After the completion of the pilot survey, the questionnaires were completed through the process of a face-to-face structured interview.

Dietary AGEs

The dietary intake of AGEs was assessed using reputable published tables that reflect the content of various foods in AGEs based on carboxymethyllysine content [8]. For foods containing AGEs between 1 and 100 kU/100 gr, the units/portions of each food consumed per week were multiplied by 1; for foods with 101 to 1,000 kU AGEs/100 gr, by 10; and for contents higher than 1,000 kU/100 gr, by 100.

Hematological/biochemical markers

Peripheral blood samples were collected from all fasting participants for subsequent analysis to check the hematological and biochemical markers. In patients, blood sampling was performed during the paracentesis of the arteriovenous access before the start of the hemodialysis. A complete blood count was performed (red blood cells, white blood cells, hematocrit, hemoglobin, platelets), as well as an analysis of the biochemical markers and, more specifically, an analysis of renal function markers such as urea, creatinine, and uric acid; glycemic profile such as fasting glucose and glycated hemoglobin (HbA1c); inflammation markers such as C-reactive protein (CRP), erythrocyte sedimentation rate, and ferritin; lipidemic profile such as total cholesterol, low-density lipoprotein (LDL) cholesterol, high-density lipoprotein (HDL) cholesterol, and triglycerides; and electrolytes such as potassium, sodium, calcium, and phosphorus. The measurements were taken at the microbiology laboratories of the hemodialysis units using Sysmex K-4500 (Toa Medical Electronics Co., Ltd., Kobe, Japan) and Siemens Advia 1800 (Siemens Healthcare Diagnostics, Inc., Tokyo, Japan) chemistry automatic hematological analyzers.

Code of ethical conduct

This research study complies with the fundamental ethical conduct principles, which govern the conduct of research. The research protocol was also approved by the Ethics Committee of the University of Peloponnese. Relevant authorization was granted by the ethical conduct committees of the hemodialysis units, in which the patients who participated in the survey are monitored, and permissions were taken by the Hellenic Data Protection Authority. The participants were informed about the aims and procedures of the study. A signed consent was obtained before the completion of the questionnaires.

Statistical analysis

A descriptive analysis of the sample (patients and control subjects) was conducted, including age, gender, and socio-economic characteristics, as well as clinical and biochemical picture of the individuals of the survey, family history, dietary habits regarding the AGEs of the dietary categories, and habits of individuals in terms of food preparation. Fisher's exact test was used for quantitative variables, one-way analysis of variance was used for parametric quantitative variables, and the Kruskal-Wallis H test was used for non-parametric variables. Correlations between the studied variables were conducted using Pearson’s Rho for parametric variables and Spearman’s Rho for non-parametric variables. Finally, a prediction analysis was performed of the clinical and biochemical characteristics and AGE scores using binomial logistic regression analysis and a receiver operating characteristic curve. Statistical analysis was conducted using SPSS v.24 (SPSS Inc., Chicago, IL, USA), and the level of significance was set at p = 0.05.

## Results

Pilot study

The general and clinical condition of patients with or without CKD did not show any change between the two measurements. From the statistical analysis of the results of the pilot survey, the satisfactory reliability (Cronbach’s alpha = 0.875) and validity (Sig. (two-tailed) > 0.05) of the tool were ascertained.

Demographic data and somatometric characteristics

The demographic data and somatometric characteristics of the respondents are given in the Tables 1 and 2.

**Table 1:** Demographic data of patients and control subjects 1. Mean ± standard deviation

Demographic data		Patients (Ν = 305), Ν (%)	Control subjects (Ν = 247), Ν (%)	Healthy control subjects (Ν = 53), Ν (%)	p-Value
Gender	Male	162 (54.2)	137 (55.7)	25 (47.2)	0.0001
	Female	137 (45.8)	109 (44.3)	28 (52.8)	
Age (years) (64.12 ± 14.77)^1^	18-44	30 (9.8)	14 (5.7)	31 (58.5)	0.0001
	45-60	72 (23.6)	71 (28.7)	13 (24.5)	
	60-75	132 (43.3)	87 (35.2)	5 (9.4)	
	75+	71 (23.3)	75 (30.4)	4 (7.5)	
Place of residence	Urban area	104 (34.7)	78 (31.6)	26 (49.1)	0.011
	Rural area	195 (65.0)	168 (68.0)	27 (50.9)	

**Table 2:** Somatometric characteristics of patients and control subjects 1. Mean ± standard deviation

Somatometric characteristics	Patients (Ν = 305)^1^	Control subjects (Ν = 247)^1^	Healthy control subjects (Ν = 53)^1^	p-Value
Body mass index (kg/m^2^)	26.50 ± 4.84	30.02 ± 4.85	27.09 ± 4.21	0.0001
Waist circumference (cm)	90.58 ± 17.66	99.22 ± 14.22	89.94 ± 15.78	0.0001

Dietary habits and intake of AGEs (per food category)

Eating habits, by category and their relative calculated total AGEs, are presented in Table 3. It was found that patients with ESRD consume smaller amounts of fruits and vegetables compared with the control groups (p = 0.0001).

Healthy subjects consume more dairy products than patients with ESRD (p = 0.003). However, the AGEs derived from dairy products are more in patients with ESRD (p = 0.0001). The increased intake of AGEs for this food category is justified by the consumption of products such as full-fat milk, cheese, and yogurt.

In the group of starchy foods, the portions consumed by patients with ESRD weekly lie between the two control groups (p = 0.003), although the increased consumption of baked and toasted bread provides patients with ESRD with more dietary AGEs (p = 0.0001).

In regard to the consumption of meat and fish products, a significantly higher intake of both portions (p = 0.0001) and AGEs (p = 0.0001) was also observed in the group of patients with ESRD compared with the control subjects. Increased AGEs intake was due to the fact that meat and fish are processed mainly by frying or charcoal grilling.

Healthy control subjects consume higher amounts of sweets, whereas the diabetic control subjects consume the lowest ones, with corresponding results also in the intake of AGEs (p = 0.0001).

In regard to junk food and charcuterie intake, it was observed that patients with ESRD consumed smaller amounts of these food categories (p = 0.018 and p = 0.0001, respectively) and, as a result, less AGEs derived from these food categories compared with the control group (p = 0.018 and p = 0.0001, respectively).

Regarding raw olive oil, the intake was found to be higher in the diabetic group and the least in the healthy control subjects (p = 0.0001).

Comparing the total AGEs that are included in the diet of the three groups, it was observed that diabetic patients have the lowest AGE intake and healthy control subjects have the highest AGE intake, with patients with ESRD lying in the middle but with high rates (p = 0.0001).

**Table 3:** Eating habits of patients and controls (by category and their relative calculated total AGEs) AGEs, advanced glycation end products *Mean ± standard deviation 1: pieces; 2: portions; 3: tablespoons

	Patients (Ν = 305)^*^	Control subjects (Ν = 247)^*^	Healthy control subjects (Ν = 53)^*^	p-Value	
Fruits^1^ per week	10.15 ± 9.29	11.73 ± 7.60	24.62 ± 16.35		0.0001	
Total AGEs	10.15 ± 9.29	11.73 ± 7.60	24.62 ± 16.35	0.0001	
Vegetables^2^ per week	13.41 ± 7.47	15.54 ± 7.19	22.43 ± 10.46	0.0001	
Total AGEs	178.45 ± 171.77	145.58 ± 104.41	153.02 ± 109.74	0.893	
Dairy products^2^ per week	11.52 ± 6.95	9.32 ± 6.45	10.75 ± 6.81	0.0001	
Total AGEs	2310.49 ± 4216.78	1063.94 ± 1973.99	3177.70 ± 4806.77	0.003	
Starch^2^ per week	21.85 ± 11.79	22.78 ± 13.51	19.02 ± 2.97	0.005	
Total AGEs	121.75 ± 80.31	85.96 ± 72.41	55.53 ± 51.20	0.0001	
Meat/fish^2^ per week	18.49 ± 10.49	4.12 ± 1.95	8.79 ± 7.92	0.0001	
Total AGEs	1827.97 ± 1046.34	403.36 ± 185.59	833.39 ± 757.25	0.0001	
Cold cuts^1^ per week	0.18 ± 0.81	0.32 ± 1.03	1.28 ± 1.97	0.0001	
Total AGEs	0.06 ± 0.24	0.14 ± 0.40	0.46 ± 0.61	0.0001	
Sauces^3 ^(mustard, mayonnaise, ketchup, etc.) per week	0.01 ± 0.11	0.02 ± 0.15	0.02 ± 0.19	0.054	
Total AGEs	98.03 ± 140.80	208.29 ± 162.33	132.17 ± 158.99	0.0001	
Sweets^1^ per week	3.12 ± 2.79	7.43 ± 11.94	22.02 ± 24.81	0.0001	
Total AGEs	16.78 ± 18.33	7.43 ± 11.94	22.02 ± 24.81	0.0001	
Snacks^1 ^(cookies, crisps, nuts, cereals, etc.) per week	3.61 ± 4.28	3.21 ± 3.90	10.77 ± 11.07	0.0001	
Total AGEs	50.76 ± 85.98	82.61 ± 149.89	140.85 ± 155.62	0.0001	
Fast food^2^ per week	2.59 ± 3.35	2.75 ± 3.20	4.92 ± 6.85	0.018	
Total AGEs	259.01 ± 335.10	274.90 ± 320.47	492.45 ± 685.24	0.018	
Canned food^1^ per week	0.27 ± 1.07	0.15 ± 0.48	0.38 ± 1.00	0.048	
Total AGEs	2.25 ± 7.92	2.18 ± 13.46	6.32 ± 30.66	0.056	
Drinks^2^ per week	12.76 ± 6.46	12.32 ± 5.81	15.47 ± 7.52	0.015	
Total AGEs	12.76 ± 6.46	12.32 ± 5.81	15.47 ± 7.52	0.015	
Alcoholic beverage^2^ per week	4.01 ± 4.99	4.09 ± 5.66	4.22 ± 5.90	0.568	
Total AGEs	4.01 ± 4.99	4.09 ± 5.66	4.22 ± 5.90	0.568	
Raw olive oil^3^ per week	22.2 ± 16.93	28.97 ± 19.83	21.4 1 ± 13.83	0.0001	
Total AGEs	222.00 ± 169.37	289.79 ± 198.36	214.15 ± 138.35	0.0001	
Total AGEs	5271.28 ± 5087.77	2786.16 ± 2511.88	5736.07 ± 5917.81	0.0001	

Hematological/biochemical markers of patients with ESRD and control subjects

The hematological and biochemical characteristics of the respondents are given in Table 4.

**Table 4:** Hematological/biochemical markers of patients and control subjects SD, standard deviation; ΗbA1c, glycated hemoglobin; CRP, C-reactive protein; ESR, erythrocyte sedimentation rate; LDL, low-density lipoprotein; HDL, high-density lipoprotein 1. Mean ± standard deviation

	Patients (Ν = 305)	Control subjects (Ν = 247)	Healthy control subjects (Ν = 53)	
COMPLETE BLOOD COUNT	Mean ± SD^1^	Mean ± SD^1^	Mean ± SD^1^	p-Value
Red blood cells (*10^3^/mm^3^)	3975.77 ± 911.06	4573.62 ± 778.97	4622.62 ± 728.35	0.0001
White blood cells (*10^3^/mm^3^)	7240.95 ± 2179.80	7506.11 ± 2294.16	6957.92 ± 2620.32	0.083
Platelets (*10^3^/mm^3^)	204.82 ± 69.59	244.35 ± 76.80	229.73 ± 74.11	0.0001
Hematocrit (%)	35.37 ± 3.48	39.63 ± 5.23	40.18 ± 3.74	0.0001
Hemoglobin (g/dL)	11.43 ± 1.21	13.85 ± 1.61	13.35 ± 1.48	0.0001
RENAL FUNCTION MARKERS	Mean ± SD^1^	Mean ± SD^1^	Mean ± SD^1^	p-Value
Urea (mg/dL)	153.07 ± 44.21	48.03 ± 28.61	27.60 ± 9.42	0.0001
Creatinine (mg/dL)	8.07 ± 2.54	1.11 ± 0.56	0.73 ± 0.31	0.0001
Uric acid (mg/dL)	6.05 ± 1.34	5.89 ± 1.87	4.61 ± 0.98	0.0001
GLYCEMIC MARKERS	Mean ± SD^1^	Mean ± SD^1^	Mean ± SD^1^	p-Value
Fasting blood glucose (mg/dL)	125.62 ± 62.17	156.77 ± 65.51	92.32 ± 12.28	0.0001
ΗbA1c (%)	6.12 ± 1.42	6.98 ± 1.32	5.00 ± 0.52	0.0001
INFLAMMATORY MARKERS	Mean ± SD^1^	Mean ± SD^1^	Mean ± SD^1^	p-Value
CRP (mg/dL)	4.21 ± 6.96	2.14 ± 1.17	1.55 ± 1.34	0.446
ESR (mm/h)	39.07 ± 25.12	25.78 ± 14.22	13.00 ± 11.01	0.005
Ferritin (ng/mL)	305.43 ± 242.98	277.34 ± 184.69	59.93 ± 53.41	0.002
LIPIDEMIC MARKERS	Mean ± SD^1^	Mean ± SD^1^	Mean ± SD^1^	p-Value
Total cholesterol (mg/dL)	162.92 ± 43.78	178.74 ± 57.53	151.40 ± 51.55	0.0001
LDL cholesterol (mg/dL)	86.22 ± 33.05	106.69 ± 34.09	99.25 ± 24.29	0.0001
HDL cholesterol (mg/dL)	48.33 ± 15.74	50.82 ± 17.31	58.90 ± 12.64	0.0001
Triglycerides (mg/dL)	185.75 ± 106.58	159.92 ± 98.10	92.70 ± 43.86	0.0001
ELECTROLYTES	Mean ± SD^1^	Mean ± SD^1^	Mean ± SD^1^	p-Value
Sodium (mmol/L)	139.95 ± 2.16	140.01 ± 2.25	139.71 ± 2.88	0.0001
Potassium (mmol/L)	5.14 ± 0.71	4.70 ± 0.59	4.41 ± 0.41	0.0001
Calcium (mmol/L)	9.01 ± 0.89	9.82 ± 1.37	9.56 ± 0.25	0.0001
Phosphorus (mmol/L)	5.06 ± 1.37	4.18 ± 2.04	3.55 ± 0.92	0.0001

Correlations

Renal function markers and dietary AGEs: Creatinine of patients with ESRD was positively correlated with the AGEs that are derived from the junk food category (r = 0.231, p = 0.00), and uric acid was positively correlated with AGEs that are derived from alcoholic beverages (r = 0.207, p = 0.004). The uric acid of healthy control subjects was positively correlated with the AGEs that are derived from the categories of junk food and alcoholic beverages (r = 0.472, p = 0.003 and r = 0.460, p = 0.004 respectively).

Glycemic profile markers and dietary AGEs: HbA1c of patients with ESRD was positively correlated with the AGEs derived from the consumption of fruits (r = 0.200, p = 0.00) and alcoholic beverages (r = 0.222, p = 0.00), and HbA1c of the control subjects with DM was positively correlated with the AGEs derived from vegetables (r = 0.202, p = 0.001) and raw olive oil (r = 0.291, p = 0.000 ), as well as with the total AGEs in their diet (r = 0.314, p = 0.000). For healthy control subjects, glucose and the AGEs that are derived from alcoholic beverages show positive correlation (r = 0.279, p = 0.043), whereas HbA1c shows a positive correlation with the AGEs that are derived from junk food (r = 0.407, p = 0.002) and a moderately negative correlation with the AGEs derived from fruits (r = -0.395, p = 0.03).

Inflammatory markers and dietary AGEs: None of the inflammatory markers was correlated with AGEs in a specific food category for patients with ESRD, but in the control subjects with DM, a negative correlation was observed between ESR and the AGEs of fruits (r = -0.472, p = 0.041). Regarding the CRP, the results of the survey show that in diabetic patients, there is a positive correlation with the AGEs of dairy products (r = 0.321, p = 0.036) and starchy foods (r = 0.344, p = 0.024), as well as total AGEs (r = 0.327, p = 0.032). For healthy control subjects positive correlation was observed between ESR and AGEs derived from dairy products, as well as total AGEs (r = 0.812, p = 0.05 and r = 0.899, p = 0.015, respectively), CRP was positive correlated with vegetable and total AGEs (r = 0.750, p = 0.05 and r = 0.857, p = 0.014, respectively).

Lipidemic profile and dietary AGEs: The AGEs that are contained in beverages and soft drinks were positively correlated with triglycerides (r = 0.231, p = 0.001) in patients with ESRD. In the diabetic patients of the survey, a negative correlation was observed between HDL and total AGEs (r = -0.224, p = 0.000). A positive correlation was observed between the total cholesterol of healthy control group and AGEs of meat products (r = 0.243, p = 0.089), LDL and AGEs of sweets (r = 0.272, p = 0.062) and triglycerides and AGEs of dairy products (r = 0.306, p = 0.031).

## Discussion

AGEs, both endogenous and exogenous, constitute a different field of study for the investigation of the pathogenesis and progression of chronic diseases such as CKD. In this context, this study attempts to investigate and correlate dietary AGEs intake with the hematological and biochemical profile of patients with ESRD undergoing hemodialysis. Among the key findings was that the patients with ESRD of this survey received extremely high amounts of AGEs through their diet.

The majority of the dietary AGE intake that was observed in patients with ERSD was in the category of meat and fish, which are mainly oven-baked, charcoal-grilled, or fried. This way of cooking changes the nutritional composition of foods, as the absorption of fat and loss of water increases the formation of AGEs [9].

For the patients with ESRD of this survey, a positive correlation of HbA1c with the consumption of fruits and alcoholic beverages and the resultant AGEs was observed, whereas for the group of control subjects with DM, a positive correlation of HbA1c with the AGEs that are derived from the category of vegetables was observed.

In hyperglycemia conditions where increased oxidative stress conditions prevail, a decreased concentration of antioxidant substances is observed and the formation of AGEs is favored [10].Fruits and vegetables are rich sources of some antioxidants such as carotenoids and vitamins A, C, and E, and their intake contributes to the decrease in the values of HbA1c. This conclusion is also reached by the survey of Sargeant et al., in which the increased consumption of fruits and vegetables was linked to the decreased value of HbA1c as opposed to the result of this survey [11].

The moderate consumption of alcohol is also correlated with a decreased incidence of DM and a decreased incidence of heart diseases in individuals with DM [12-13]. The results of this survey among patients with ESRD showed that the consumption of a moderate amount of alcohol, mostly in the form of wine, is correlated with increased HbA1c levels, and this is in contrast with the survey of Hong et al., who observed that the intake of alcoholic beverages was positively correlated with the drop in HbA1c levels, although in healthy individuals and not in patients with ESRD under hemodialysis [14]. It should be noted, however, that the high consumption of alcohol is linked with the formation of AGEs, insulin resistance, and secondary DM complications since acetaldehyde, one of the main metabolites of alcohol, when in excess, turns into a toxic substance, forming addition compounds with DNA, lipids, and proteins. Alcohol also tends to speed up the oxidative stress by reducing antioxidants such as vitamins A, C, and E, taking thus part in the formation of AGEs [15].

Although the consumption of junk foods and AGEs that are derived from this category was less in patients with ESRD of this survey compared with the control groups, it emerges from the results that these AGEs are positively correlated with creatinine. Creatine in meat is turned into creatinine during cooking [16]. Creatine increases the levels of C-pentosidine that is frequently detected in the plasma of patients with ESRD who undergo hemodialysis. Pentosidine, which is one of the most important AGEs structures, and its levels in plasma reflect the severity of diabetic nephropathy and CΚD is formed by the reaction of ribose with lysine and arginine. In renal dysfunction conditions such as CΚD, creatine levels in the plasma are increased and are much higher than arginine levels. As a result, creatine takes the place of arginine during the formation of pentosidine, resulting in C-pentosidine [17].

Uric acid was positively correlated with the consumption of alcoholic beverages and AGEs that are derived from this category in the healthy control subjects of this survey. Uric acid is the end product of purine metabolism, and, therefore, the consumption of foods that are rich in purines contributes to an increase in the levels of uric acid. Increased uric acid levels are associated with systematic inflammation and endothelial dysfunction. Ethanol increases the concentration of purines (hypoxanthine and xanthine) in plasma since it speeds up the decomposition of adenine, which is the nitrogen base of nucleotides and belongs to purines. Ethanol also increases the level of galactic acid in the blood, which inhibits the secretion of uric acid [18-19].

For the category of patients with ESRD in this survey, a positive correlation was recorded between the consumption of soft drinks and AGEs that are derived from them, and triglycerides. Soft drinks are a big source of carbohydrates. Excess dietary carbohydrate content promotes the composition of lipids in the kidney, turning carbohydrates into triglycerides for long-term energy storage [20]. The positive correlation between the increased consumption of carbohydrates and increased triglyceride levels was confirmed by the study of Min et al. [21]. The consumption of soft drinks with high fructose content along with meals resulted in increased triglyceride levels compared with soft drinks rich in glucose, as was recorded in the survey of Teff et al. [22], and the same conclusion was also reached by the survey of Bantle et al among 24 healthy adults (12 males and 12 females), in which dietary fructose was correlated with increased concentrations of plasma triglycerides in the males of the survey [23].

However, this study has some limitations. The conduct of the survey on an even bigger sample of both patients and control subjects would support the reliability of the results more, and the correlation would be useful with additional factors as well, such as concomitant diseases and the number of years that the patients of the survey undergo hemodialysis.

## Conclusions

In general, the data of the survey support the assumption that dietary AGEs play an important role in the progression of CKD as well as in the adverse prognosis of patients with ESRD who undergo hemodialysis. The consumption of foods that contain high levels of AGEs, but, most of all, the method by which foods are processed or cooked, even those that are healthy options, for example, fruits, vegetables, cereals, and fish, disrupt the hematological markers of these patients and place an additional burden on the progression of their vascular disease that is the main cause of their morbidity and mortality, concomitant diseases, and the number of years that the patients of the survey undergo hemodialysis.
